# *Prdm9*, a Major Determinant of Meiotic Recombination Hotspots, Is Not Functional in Dogs and Their Wild Relatives, Wolves and Coyotes

**DOI:** 10.1371/journal.pone.0025498

**Published:** 2011-11-10

**Authors:** Violeta Muñoz-Fuentes, Anna Di Rienzo, Carles Vilà

**Affiliations:** 1 Estación Biológia de Doñana EBD-CSIC, Sevilla, Spain; 2 Department of Population and Conservation Biology, Evolutionary Biology Center, Uppsala University, Uppsala, Sweden; 3 Department of Human Genetics, University of Chicago, Chicago, Illinois, United States of America; Oklahoma Medical Research Foundation, United States of America

## Abstract

Meiotic recombination is a fundamental process needed for the correct segregation of chromosomes during meiosis in sexually reproducing organisms. In humans, 80% of crossovers are estimated to occur at specific areas of the genome called recombination hotspots. Recently, a protein called PRDM9 was identified as a major player in determining the location of genome-wide meiotic recombination hotspots in humans and mice. The origin of this protein seems to be ancient in evolutionary time, as reflected by its fairly conserved structure in lineages that diverged over 700 million years ago. Despite its important role, there are many animal groups in which *Prdm9* is absent (e.g. birds, reptiles, amphibians, diptera) and it has been suggested to have disruptive mutations and thus to be a pseudogene in dogs. Because of the dog's history through domestication and artificial selection, we wanted to confirm the presence of a disrupted *Prdm9* gene in dogs and determine whether this was exclusive of this species or whether it also occurred in its wild ancestor, the wolf, and in a close relative, the coyote. We sequenced the region in the dog genome that aligned to the last exon of the human *Prdm9*, containing the entire zinc finger domain, in 4 dogs, 17 wolves and 2 coyotes. Our results show that the three canid species possess mutations that likely make this gene non functional. Because these mutations are shared across the three species, they must have appeared prior to the split of the wolf and the coyote, millions of years ago, and are not related to domestication. In addition, our results suggest that in these three canid species recombination does not occur at hotspots or hotspot location is controlled through a mechanism yet to be determined.

## Introduction

Meiotic recombination has been the focus of much attention because it is a fundamental process needed for the correct segregation of chromosomes during meiosis in sexually reproducing organisms, and it may profoundly affect population genetic diversity by unlinking genes located on the same chromosome (e.g. [1], [2]; and references therein). In humans, 80% of crossovers are estimated to take place in 10%–20% of the genome sequence, which contain the so-called recombination hotspots [3]. The location of these hotspots was found not to be conserved across closely related species, such as human and chimpanzee [4]–[6]. The increasing availability of bioinformatic and genomic tools to study recombination have contributed to the recent explosion of literature on this topic in order to understand the fundamentals of how this process takes place and its consequences. A protein called PRDM9 (also known as Meisetz) has been pinpointed as playing a role in the determination of recombination hotspots and its study has recently attracted much interest. However, many questions remain unanswered about its molecular mode of action.

**Figure 1:**
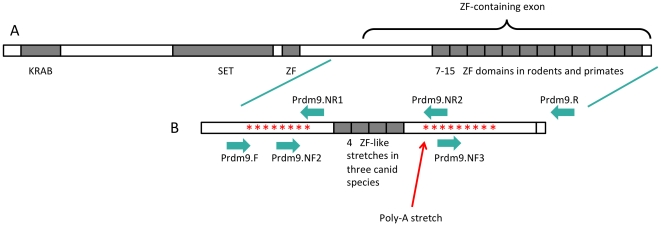
General structure of PRDM9 in rodents and primates and position of the primers used and stop codons found in this study in dogs, wolves and coyotes. (A) PRDM9 as described for most metazoans, in particular primates and rodents [8], [9], [47]. (B) Region sequenced in this study aligning to the last exon of humans as shown by the ECR browser (see text for details); position of the primers is indicated by arrows and position of the stop codons is represented by asterisks (for detailed information, refer to the text and tables).

PRDM9 was found to be expressed in germ-line cells during meiosis in mice [7] and it was later shown to play an essential role in meiosis and speciation in a number of metazoan species [8]. Most recently, evidence has been provided that it is a determinant of sequence-specific meiotic recombination hotspots in humans and mice [9]–[12]. The PRDM9 protein in human and mice has three functional domains: (1) an N-terminal KRAB domain typically associated with zinc finger proteins and involved in protein-protein interactions and transcriptional repression; (2) a central SET domain with histone methyl transferase activity (thus capable of trimethylating H3K4 and consequently altering chromatin configuration); and (3) multiple C_2_H_2_ zinc finger (ZF) domains in tandem near the C-terminal part of the protein [13]–[16] (Fig. 1). The ZF array selectively binds to specific DNA sequences, and amino acid substitutions in the ZFs as well as polymorphism in their number affects the DNA sequences that the protein recognizes [8], [9], [17]. PRDM9 is a rapidly evolving protein due to the instability derived from the minisatellite structure of the ZF array, thus conferring a capacity for different alleles to quickly emerge, which will bind to a variety of DNA sequences. Multiple studies have suggested that this gene has undergone strong positive selection [8], [11], [17] and its rapid evolution implies changes in the DNA sequence patterns that different PRDM9 alleles may recognize, with the potential to affect hotspot location genome-wide [9], [12]. This may explain the occurrence of hybrid sterility in individuals resulting from crossing closely related mice species [8], [17]–[19] or the different location of human hotspots compared to those of chimpanzees' [11]. In addition, it has been observed that the number of ZF repeats and single amino acid substitutions affect the activation, enhancement and appearance of recombination hotspots in humans [20], [21].

In a study of ZF sequences in a diverse panel of 35 metazoan species spanning about 700 million years of evolution, it was proposed that, despite *Prdm9*'s important role at meiosis, it had acquired several disruptive mutations in the dog, *Canis familiaris* [8]. Dogs have a unique evolutionary history. Through domestication and strong artificial selection, humans have created the most phenotypically diverse vertebrate species, and the genetic mechanisms underlying this diversity are only partially understood. Dog breed isolation started a few hundred years ago, but domestication and selection of specific phenotypic and behavioural characters began tens of thousands of years ago [22]–[25]. The morphological [26], [27], behavioural and physiological [28] differences among dogs are larger than the differences observed across the entire family Canidae, which includes about 35 species such as raccoon dogs, foxes, wolves, jackals and coyotes, and that have evolved over 15 million years. Several mechanisms have been hypothesized to explain the large phenotypic diversity found in dogs, including the relaxation of selective forces acting upon the dog genome as compared to the wolf genome [29], [30], modifications in structural genes (e.g. [31]–[36]), the presence of repetitive and/or SINE elements that could affect the function of genes [37], [38] or an elevated recombination rate in dogs as compared to wolves. Chiasma frequencies for domestic animals, especially for the dog, were observed to be larger than expected according to their age to maturity [39] and, additionally, recombination rates have been observed to be variable between cattle families, suggesting that this trait is heritable and susceptible of being selected [40]. High recombination rates would allow novel trait combinations to emerge, although it is not clear how much diversity was present in the ancestral species. Therefore, we decided to study the diversity of *Prdm9* and its functional status in the dog and compare it to the orthologue in the wild ancestor, the grey wolf (*Canis lupus*), and in a close wild relative, the coyote (*Canis latrans*).

## Materials and Methods

We sequenced the region aligning to the human's last exon of *Prdm9* from the genomic DNA of four dogs, 17 wolves and two coyotes. Dogs were either purebred (German shepherd, *n* = 1) or mongrels/crossbred (*n* = 3). Wolves had a variety of geographical origins (Canada, North West Territories, *n* = 3; Canada, British Columbia, *n* = 1; Italy, *n* = 3; Finland, *n* = 4; Spain, *n* = 1; Sweden, *n* = 3; USA, a captive population in Minnesota, *n* = 2). The coyotes were from Colorado and Nebraska (USA). We used the alignment between human and dog genome sequences available at the ECR browser to locate the areas surrounding the last exon of the *Prdm9* gene in humans and the corresponding region in the dog. We used this information to download the corresponding sequence of the dog genome from the UCSC browser and then designed primers that would amplify a region of about 2000-bp that was expected to contain the entire ZF domain. We also designed four additional internal primers and used all six of them to sequence this region (Fig. 1, Table 1). DNA was amplified using the LongRange PCR Kit (QIAGEN, Hilden, Germany) in 35-µl reactions containing 1× buffer (10× LongRange PCR Buffer), 0.5 mM dNTPs each (10 mM dNTP mix as provided), 0.4 µM of each primer CanPrdm9.F and CanPrdm9.R, 1.4 U of Taq polymerase (LongRange PCR Enzyme Mix) and 10–100 ng genomic DNA. PCRs were performed in an ABI 2700 thermal cycler (Applied Biosystems) or MJ Research DNA Engine Tetrad with an initial denaturation step of 93°C for 3 min followed by 35 cycles of 93°C for 15 s, 58°C for 30 s and 68°C for 2 min and a final extension of 68°C for 7 min. DNA-free controls were included in all cases to monitor for potential contamination.

**Table 1:** Primers used to amplify and sequence in dogs, wolves and coyotes the region aligning to the human last exon of the *Prdm9* gene.

Primer Name	Sequence
CanPrdm9.F	AGAGAAGCTGCCTCTGATGC
CanPrdm9.R	CTGGACCCTTTTGCTTTCAG
CanPrdm9.NR1	AATTTGCCTGTGTCCTCTGG
CanPrdm9.NF2	GCAGGCTCACAGAAATTGAA
CanPrdm9.NR2	TGAAGCCTCTAAGTGTGTCCTC
CanPrdm9.NF3	GGACACACTTAGAGGCTTCATC

PCR products were run in 1% agarose gels and were excised and purified using the QIAquick Gel Extraction Kit (QIAGEN, Hilden, Germany) following the manufacturer's instructions. Both strands of each PCR product were then sequenced with the six primers (Table 1) and reaction products were separated in an automated sequencer (ABI 3730xl DNA Analyzer; Applied Biosystems). Sequences from multiple PCRs were concatenated and edited using Sequencher ver. 4.6 (Gene Codes Corporation, Ann Arbor, MI, USA), and were then aligned by eye using Se-Al ver. 2.0a11 Carbon [41] (Accession Numbers: HE590859–HE590881). We then used Sequencher, Se-Al and the TIME Sequence Editor [42] to translate the DNA to all reading frames using the standard genetic code. PHASE 2.1 [43], [44] was used to construct haplotypes.

## Results

Several lines of evidence are consistent with the idea that the region we sequenced is an orthologue to *Prdm9* and not a paralogue. Both the *Prdm9* gene and the protein have been well established for humans and the house mouse. Since primates and rodents are more closely related to each other than to carnivores [45], the sequence information from either species is equally appropriate for comparison with canids, and we decided to take the human sequence as reference. First, we used the ECR browser to find the region in the dog that aligned to the human last exon of *Prdm9* (see Materials and Methods). Second, we blasted one of the dog sequences (the reference sequence, CanFam2) and found that the most similar matching sequence was to *Prdm9* genes in *Bos taurus*, *Homo sapiens*, *Macaca mulatta*, *Mus musculus*, *Nomascus leucogenys*, *Pan troglodytes* and *Pongo abelii*. The maximum identity ranged between 94% and 83% and the part of the query sequence that was covered ranged between 48% and 22%, comprising the part of the sequence ranging from position ∼200 to ∼1200 (Table 2). The next most similar matching sequence was to the predicted *Prdm7* of *Ailuropoda malnoleuca* (the giant panda), covering only 17% of the query sequence (90% similarity), corresponding to positions 168–505 (Table 2). Because the canid sequences we obtained were almost identical (Table 2), we blasted just one of them. Third, we took the sequence of the confirmed *Prdm9* gene and PRDM9 protein in humans and did a blast, a protein blast and a tblastn against the dog genome and found high similarity only to ZF proteins. Finally, multiple paralogues of *Prdm9* have been found in primates, ruminants and monotremes [46], [47], but have not been reported for other species, including the dog [47].

**Table 2:** Polymorphic nucleotide positions found in dogs, wolves and coyotes in the region of the *Prdm9* gene sequenced.

Individual	Breed/Geographical origin^a^	Data type	N (position in sequence)^c^	75	91	126	231	330	560	713	769	964	966	967	976	986	988	989	990	991	1034	1157	1177	1183	1208	1219	1306	1307	1308	1314	1332	1470	1555	1582
Dog (CanFam2)	Boxer	Reference		T	T	G	T	T	G	G	C	A	G	A	G	G	A	G	G	A	G	C	T	C	C	C	–	A	A	A	A	C	A	T
Dog_1	German shepherd	Experimental		A	.	A	G	C	.	.	.	.	.	.	.	.	.	.	.	.	.	T	.	A	.	T	.	.	.	.	.	.	.	.
Dog_2	Mix breed	Experimental		W	.	A	K	Y	.	.	.	.	.	.	.	.	.	.	.	.	.	Y	.	.	.	Y	.	.	.	.	.	.	.	.
Dog_3	Mix breed	Experimental		A	.	A	G	C	.	.	.	.	.	.	.	.	.	.	.	.	.	T	.	A	.	T	A	.	.	.	.	.	.	.
Dog_4	Mix breed	Experimental	Pos. 1–17	.	.	A	.	.	.	R	.	.	.	.	.	.	.	.	.	.	.	.	.	.	.	.	.	.	.	.	.	.	.	.
Wolf_1	Sweden	Experimental		.	.	A	.	.	.	.	.	.	.	.	.	.	.	.	.	.	.	.	.	.	.	.	.	.	.	.	.	.	.	.
Wolf_2	Sweden	Experimental		.	.	A	.	.	.	.	.	.	.	.	.	.	.	.	.	.	.	.	.	.	.	.	.	–	–	.	.	.	.	.
Wolf_3	Sweden	Experimental		.	.	A	.	.	.	.	.	.	.	.	.	.	.	.	.	.	.	.	.	.	.	.	.	–	–	.	.	.	.	.
Wolf_4	Spain	Experimental		.	.	A	.	.	.	.	.	W	R	M	.	R	R	.	.	.	.	.	Y	.	Y	.	.	.	.	.	.	.	.	.
Wolf_5	Finland	Experimental		.	.	A	.	.	.	.	.	.	.	.	.	.	.	.	.	.	.	.	.	.	.	.	.	–	.	.	.	.	.	.
Wolf_6	Finland	Experimental	Pos. 1–17	A	Y	A	G	C	.	.	.	.	.	.	.	R	.	.	.	.	R	T	.	A	.	T	.	.	.	.	.	.	.	.
Wolf_7	Finland	Experimental	Pos. 1–20	.	.	A	.	.	.	.	.	.	.	.	.	.	.	.	.	.	.	.	.	.	.	.	A	.	.	.	.	.	.	.
Wolf_8	Finland	Experimental		W	.	A	K	Y	.	.	.	.	.	.	.	.	.	.	.	.	R	Y	.	M	.	Y	.	.	.	.	.	.	.	.
Wolf_9	Italy	Experimental		.	.	A	.	.	.	.	.	.	.	.	.	.	.	.	.	.	.	.	.	.	.	.	.	.	.	.	.	.	.	.
Wolf_10	Italy	Experimental	Pos. 1–17	.	.	A	.	.	.	.	.	.	.	.	.	.	.	.	.	.	.	.	.	.	.	.	.	–	.	.	.	.	.	.
Wolf_11	Italy	Experimental		.	.	A	.	.	.	.	.	.	.	.	.	.	.	.	.	.	.	.	.	.	.	.	.	–	.	.	.	.	.	.
Wolf_12	North West Territories	Experimental	Pos. 1–17	W	.	A	K	Y	R	.	.	.	.	.	.	.	.	.	.	.	.	.	.	.	.	.	.	.	.	.	.	.	.	.
Wolf_13	North West Territories	Experimental		.	.	A	.	.	.	.	.	.	.	.	.	.	.	.	.	.	.	.	.	.	.	.	.	.	.	.	G	.	.	.
Wolf_14	North West Territories	Experimental		.	.	A	.	.	.	.	.	.	.	.	R	.	.	.	.	.	.	Y	.	.	.	Y	.	–	.	G	.	Y	R	W
Wolf_15	Captive, North America	Experimental		.	.	A	.	.	.	.	.	.	.	.	.	.	.	.	.	.	.	.	.	.	.	.	.	–	.	G	.	Y	R	W
Wolf_16	Captive, North America	Experimental		.	.	A	.	.	.	.	.	.	.	.	.	.	.	.	.	.	.	.	.	.	.	.	.	–	.	G	.	T	G	A
Wolf_17	British Columbia	Experimental	Pos. 1–17	.	.	A	.	.	.	.	.	.	.	.	.	.	.	.	.	.	.	.	.	.	.	.	.	.	.	.	.	.	.	.
Coyote_1	Colorado	Experimental		.	.	A	.	.	.	.	.	.	.	.	.	.	.	–	–	–	.	.	.	.	.	.	.	.	.	.	.	T	G	A
Coyote_2^b^	Nebraska	Experimental	Pos. 990–1307	.	.	A	.	.	.	.	Y	.	.	.	.	.	.	N	N	N	N	N	N	N	N	N	N	N	.	.	.	T	G	A
**Features**							Align to human and cat.^d^				Four ZF-like regions.^e^																Poly-A stretch.^f^							

a Breed in the case of dogs and the geographical origin in the case of wolves and coyotes is indicated.

b This individual seems to have a 3-bp deletion in one allele but not in the other,which made it impossible to read part of the sequence.

c Sequence for which there is no data (N).

d Positions 245–654. See text and Table S2 for more information.

e Refer to the text and Tables 3 and S1 for more information.

f Uncertainty in the nucleotide sequence due the prsence of a poly-A stretch. More information in the text.

The sequences we obtained ranged between 1,568 and 1,886 base pairs per individual (Table 2). In particular, we were not able to clearly read positions 1–17 for one dog and four wolves, and positions 1–20 for one wolf. For one coyote it was not possible to read the sequence between positions 990 and 1307 because this individual seemed to have a 3-bp deletion in one allele as compared to the other. In addition, all individuals had a poly-A stretch starting at position 1306 containing 12 to 15 As, although some uncertainty may exist as to the exact number of nucleotides in this region due to polymerase slippage during amplification. Ignoring the poly-A stretch, 28 variable positions were identified.

The last exon of *Prdm9* has been reported to confer functionality to the protein, as the C_2_H_2_ ZF domains are located here, and in particular positions -1, 3 and 6 in each one of them, act as DNA-binding sites [9]. We found that the region we sequenced in these three canid species has acquired several mutations that may result in a protein that is non functional, as suggested by several lines of evidence.

First, we aligned the region we sequenced in dogs, wolves and coyotes to the sequences of 15 species of mammals that were reported to contain a conserved region and to be located in the last exon of *Prdm9* (*Homo sapiens*, *Pan pygmaeus* and *Pan troglodytes*) or its last-exon candidate (the remaining 12 species) [17]. Whereas no stop codons were found in those 15 mammalian species, multiple stop codons were found in the dogs, wolves and coyotes at the same positions across the three species (Fig. 1).

Second, for the same species [17] we aligned the region reported to be conserved across them to the region in dogs, wolves and coyotes that aligned to it. This region is upstream of the ZF domain, in the ZF-containing exon. We chose to compare the canids to a cat sequence, the phylogenetically closest relative among those reported by [17], and to a human sequence taken as reference. While no stop codons were observed in either the human or the cat sequence, eight stop codons were found in dogs, wolves and coyotes, and all mutations were shared across the three species (Fig. 1; Table S2). The stop codons corresponded to TAA in positions 40, 87 and 139, to TAG in positions 109 and 133, and TGA in positions 110, 121 and 135 (Tables S2). If we ignored the presence of the stop codons and compared the amino acid sequences across the three canid species, they had almost identical sequences, with only two substitutions that would be non-synonymous at positions 29 and 106 (Tables S2). One substitution was the result of a variable second codon position (position 330 in Table 2) that resulted in either an isoleucine (ATC) in two dogs, 13 wolves and the two coyotes, threonine (ACC) in two dogs and one wolf (Finland), and both (AYC) in one dog and two wolves (Finland and North West Territories). The other substitution was present in a single wolf from the North West Territories and corresponded to a change in a first codon position that coded for methionine in one allele and valine in the other (position 976 in Table 2).

Third, we checked for the presence of ZFs in the three canid species. Previous studies have shown that ZFs in the *Prdm9* gene are of the type C_2_H_2_ [8], [9], [17], the sequence motif of which is C–X_2,4_–C–X_12_–H–X_3,4,5_–H–X. Most of the ZF sequences that were found in 35 metazoan species were complete (28 codons) and complied with the C_2_H_2_ structure, the sequence of which was C–R–E–C–X_12_–H–X_3_–H–T–G–E–K–P–Y–V [8]. In a sample of rodents and primates, the number of ZFs in the *Prdm9* ZF domain varied between 7 to12 and 9 to15, respectively [8]. In dogs, wolves and coyotes we identified only four ZF-like stretches (Table 3), almost identical across the three species (Table S1). However, if the whole sequence would be translated into a protein as described above, the sequence motif C–X_2,4_–C–X_12_–H–X_3,4,5_–H–X would not appear. The first ZF-like stretch we identified complied with the previously reported sequence for other metazoans, had 28 codons in the three canids (i.e., it was complete) and was identical in all individuals both at the nucleotide and the amino acid level, except for the presence of a stop codon (TGA) in one of the alleles of a coyote. The second ZF-like stretch was again identical across the three canids both in terms of the nucleotide and the amino acid sequences, but was one nucleotide shorter and the resulting amino acid sequence did comply with the C_2_H_2_ structure. The third ZF-like stretch had 28 codons and the C_2_H_2_ structure in all dogs and wolves, but the coyote for which we had data was one amino acid shorter due to a 3-bp deletion in this area, and so it was 27-codon long. Lastly, the fourth ZF-like stretch complied with the C_2_H_2_ structure in all dogs, wolves and the coyote for which we had data. After these, there were no more ZF-like sequences, but several additional stop codons were observed (Fig. 1).

**Table 3:** Four C_2_H_2_ ZF-like regions (denoted 1, 2, 3 and 4) found in the three canid species studied (dogs, wolves and coyotes); for comparison, a ZF from the cat and the human are included.

Species	Source	1	2	3	4	5	6	7	8	9	10	11	12	13	14	15	16	17	18	19	20	21	22	23	24	25	26	27	28
HomoSapiens03_ZnFinger	Oliver *et al.* (2009)	**C**	R	E	**C**	G	R	G	F	S	W	K	S	H	L	L	I	**H**	Q	R	I	**H**	T	G	E	K	P	Y	V
FelisCatus02_ZnFinger	Oliver *et al.* (2009)	**C**	R	E	**C**	G	R	G	F	T	Q	R	S	N	L	F	R	**H**	Q	R	T	**H**	T	G	E	K	P	Y	V
ZF-like Block1_CanisFamiliaris01	This study	**C**	R	E	**C**	G	R	G	F	I	H	R	T	N	L	I	I	**H**	Q	R	T	**H**	T	G	E	K	P	Y	V
ZF-like Block1_CanisLupus01	This study	**C**	R	E	**C**	G	R	G	F	I	H	R	T	N	L	I	I	**H**	Q	R	T	**H**	T	G	E	K	P	Y	V
ZF-like Block1_CanisLatrans01	This study	**C**	R	E	**C**	G	R	G	F	I	H	R	T	N	L	I	I	**H**	Q	R	T	**H**	T	G	E	K	P	Y	V
ZF-like Block1_CanisLatrans02	This study	**C**	R	E	**C**	G	*	G	F	I	H	R	T	N	L	I	I	**H**	Q	R	T	**H**	T	G	E	K	P	Y	V
ZF-like Block2_CanisFamiliaris01	This study	**C**	R	E	**C**	G	Q	A	L	Y	R	G	Q	I	S	A	Y	**I**	R	G	H	**T**	Q	G	R	S	P	M	
ZF-like Block2_CanisLupus01	This study	**C**	R	E	**C**	G	Q	A	L	Y	R	G	Q	I	S	A	Y	**I**	R	G	H	**T**	Q	G	R	S	P	M	
ZF-like Block2_CanisLatrans01	This study	**C**	R	E	**C**	G	Q	A	L	Y	R	G	Q	I	S	A	Y	**I**	R	G	H	**T**	Q	G	R	S	P	M	
ZF-like Block3_CanisFamiliaris01	This study	**C**	R	E	**C**	G	R	G	F	T	Q	R	S	T	L	N	E	**H**	Q	R	T	**H**	T	E	E	K	P	Y	V
ZF-like Block3_CanisLupus01	This study	**C**	R	E	**C**	G	R	G	F	T	Q	R	S	T	L	N	E	**H**	Q	R	T	**H**	T	E	E	K	P	Y	V
ZF-like Block3_CanisLupus02	This study	**C**	R	E	**C**	G	R	G	F	T	Q	R	S	T	L	I	T	**H**	Q	R	T	**H**	T	G	E	K	P	Y	V
ZF-like Block3_CanisLupus03	This study	**C**	R	E	**C**	G	R	G	F	T	Q	R	S	T	L	N	E	**H**	Q	K	T	**H**	T	E	E	K	P	Y	V
ZF-like Block3_CanisLatrans01	This study	**C**	R	E	**C**	G	R	G	F	T	Q	R	S	T	L	N	E	**H**	Q	R	T	**H**	T	E	–	K	P	Y	V
ZF-like Block4_CanisFamiliaris01	This study	**C**	R	E	**C**	G	R	S	F	T	R	R	S	T	L	I	T	**H**	Q	R	T	**H**	T	G	E	K	P	Y	V
ZF-like Block4_CanisLupus01	This study	**C**	R	E	**C**	G	R	S	F	T	R	R	S	T	L	I	T	**H**	Q	R	T	**H**	T	G	E	K	P	Y	V
ZF-like Block4_CanisLatrans01	This study	**C**	R	E	**C**	G	R	S	F	T	R	R	S	T	L	I	T	**H**	Q	R	T	**H**	T	G	E	K	P	Y	V

Additionally, in the three canid species a poly-A stretch was present that varied between 12 and 15 As in length, although some uncertainty remains as to the exact number, likely due to polymerase slippage during amplification. This poly-A stretch was not observed in other species for which *Prdm9* has been sequenced.

## Discussion

During the domestication process, the dog experienced a dramatic bottleneck and a relaxation in the selective forces that resulted in a faster accumulation of non-synonymous substitutions [29], [30]. We hypothesized that the domestication process may have resulted also in changes in the mode and rate of recombination. However, our results suggest that the dog did not acquire disruptive mutations in the last exon of *Prdm9* gene during the domestication process or later, given the fact that this gene is also disrupted in the wolf, from which the dog derives, and a close living relative, the coyote. The sequences for this particular region were almost identical across the three species. It then becomes an interesting question whether other canids also possess a disrupted *Prdm9* and when the disruptions first arose.

PRDM9 has been identified as a gene controlling the location of recombination hotspots in humans and mice [9]. In particular, the last exon of *Prdm9* seems to confer important functionality to the protein as a domain upstream of the ZF domain is conserved across several mammal species [17] and the C_2_H_2_ ZF domains located here act as DNA-binding sites [9]. Moreover, it has been observed that minor differences in the ZF domains as small as one amino acid substitution can deactivate, enhance or cause the appearance of a recombination hotspot in humans [20]. Our results suggest that the last exon of *Prdm9* has accumulated several disruptive mutations in dogs, wolves and coyotes and, consequently, the resulting protein may be non-functional. If this is the case, it becomes an intriguing question whether these three canid species have recombination hotspots and, if they have, whether there is a different mechanism not involving PRDM9 to control their location. In sexually reproducing organisms recombination is an essential process needed for the correct segregation of the chromosomes during meiosis [48] and it is known that, in mammals, recombination tends to occur at specific regions called recombination hotspots that are 1–2 kb long, separated from each other by tens of kilobases where recombination is essentially lacking [2], [49], [50]. Three types of factors have been suggested to control the location of recombination hotspots: DNA sequence motifs (e.g. [51], [52]), epigenetic mechanisms (e.g. [53], [54]) and trans-acting loci (e.g. [55]). Recently, many studies have focused on *Prdm9* (see the Introduction and references therein), likely because “These characteristics of PRDM9 neatly wrap genetic, epigenetic, and trans-acting factors known to influence recombination into one intriguing package” [56], page 1.

Despite its important role, *Prdm9* is absent in sauropsids (birds, lizards) and amphibians, but seems to be fairly conserved and functional in other metazoans diverging as much as 700 million years ago [8], [47]. The mutations we found in these three canid species were shared across individuals and so the mutations must have happened several million years ago, before the split between wolves and coyotes. Although PRDM9 appears to be a major regulator of hotspots in humans and other metazoans, we are still far from fully understanding how recombination hotspots are controlled and whether other trans regulatory factors exist [20]. For example, several studies indicate that in addition to the polymorphisms in PRDM9, polymorphisms at the *RNF* gene and an inversion on chromosome 17 [57], [58] appear to influence recombination, but their impacts are modest [21]. Notably, *Prdm9* −/− mice spermatocytes still have detectable double-strand breaks [7], and trans-acting factors responsible for hotspot location have been mapped in inbred lines of mice [59], [60]. Therefore, it is likely that there are other factors controlling for meiotic recombination hotspot specification in animals with sexual reproduction [20], [47], [56], [61].

In conclusion, our results suggest that if in fact this gene is not functional in these three canid species, recombination does not occur at hotspots or hotspot location is controlled through a different gene or mechanism yet to be determined. Alternatively, hotspot locations are mediated by *Prdm9* in ways that are different from those described for other organisms. Notably, because the mutations are shared between the domestic and two wild canids, we conclude that domestication was not associated to changes in the functionality of PRDM9. Whether recombination is controlled by the same gene or a different gene with a similar action or by a different type of mechanism is still to be determined and warrants further investigation.

## Supporting Information

Table S1 The four C_2_H_2_ ZF-like regions found in the three canid species studied (dogs, wolves and coyotes).
(DOC)

Table S2 Translation to amino acids of the region in dogs, wolves and coyotes aligning to the PRDM9 region identified by [17] as conserved across 15 mammal species.
(DOC)
