# Supplementary material for: Prdm9, a Major Determinant of Meiotic Recombination Hotspots, Is Not Functional in Dogs and Their Wild Relatives, Wolves and Coyotes
Source: PLoS One. 2011 Nov 10;6(11):e25498. doi: 10.1371/journal.pone.0025498 (PMC3213085; doi:10.1371/journal.pone.0025498)

***Prdm9*, a Major Determinant of Meiotic Recombination Hotspots, Is Not Functional in Dogs and Their Wild Relatives, Wolves and Coyotes**

**Violeta Muñoz-Fuentes, Anna Di Rienzo, Carles Vilà**

**SUPPLEMENTARY INFORMATION**

**Table S1. The four C_2_H_2_ ZF-like regions found in the three canid species studied (dogs, wolves and coyotes).**

Vertical lines separate each C_2_H_2_ ZF-like region. An asterisk indicates a stop codon. Grey shading indicates a difference in the amino acid sequence. Letters "a" and "b" following the individuals name indicate that that individual had at least one SNP; haplotypes were identified using PHASE. The sign "–" indicates no nucleotide data was available for that individual at that particular position (see Table 2 and text).


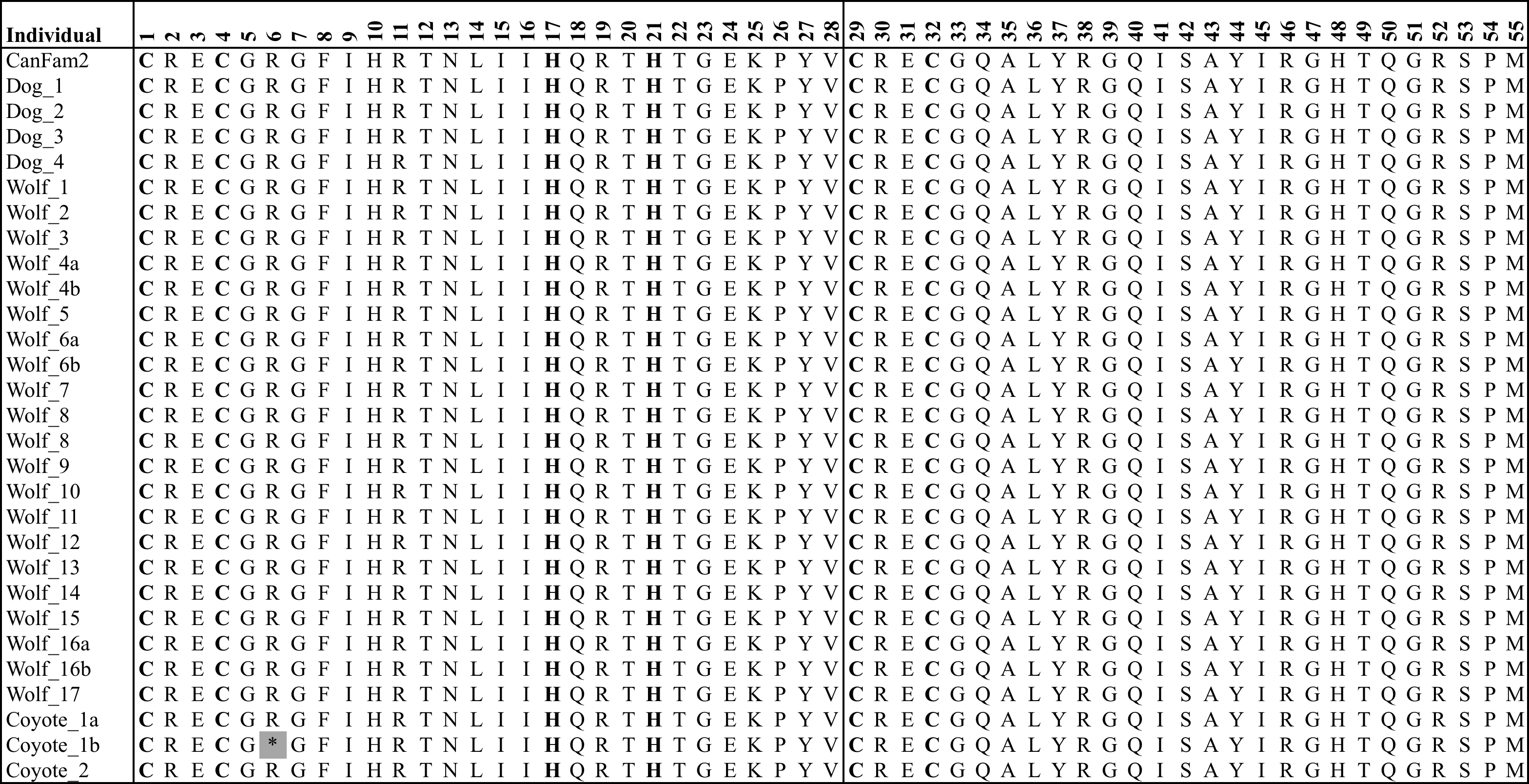


**Table S1 (continued)**


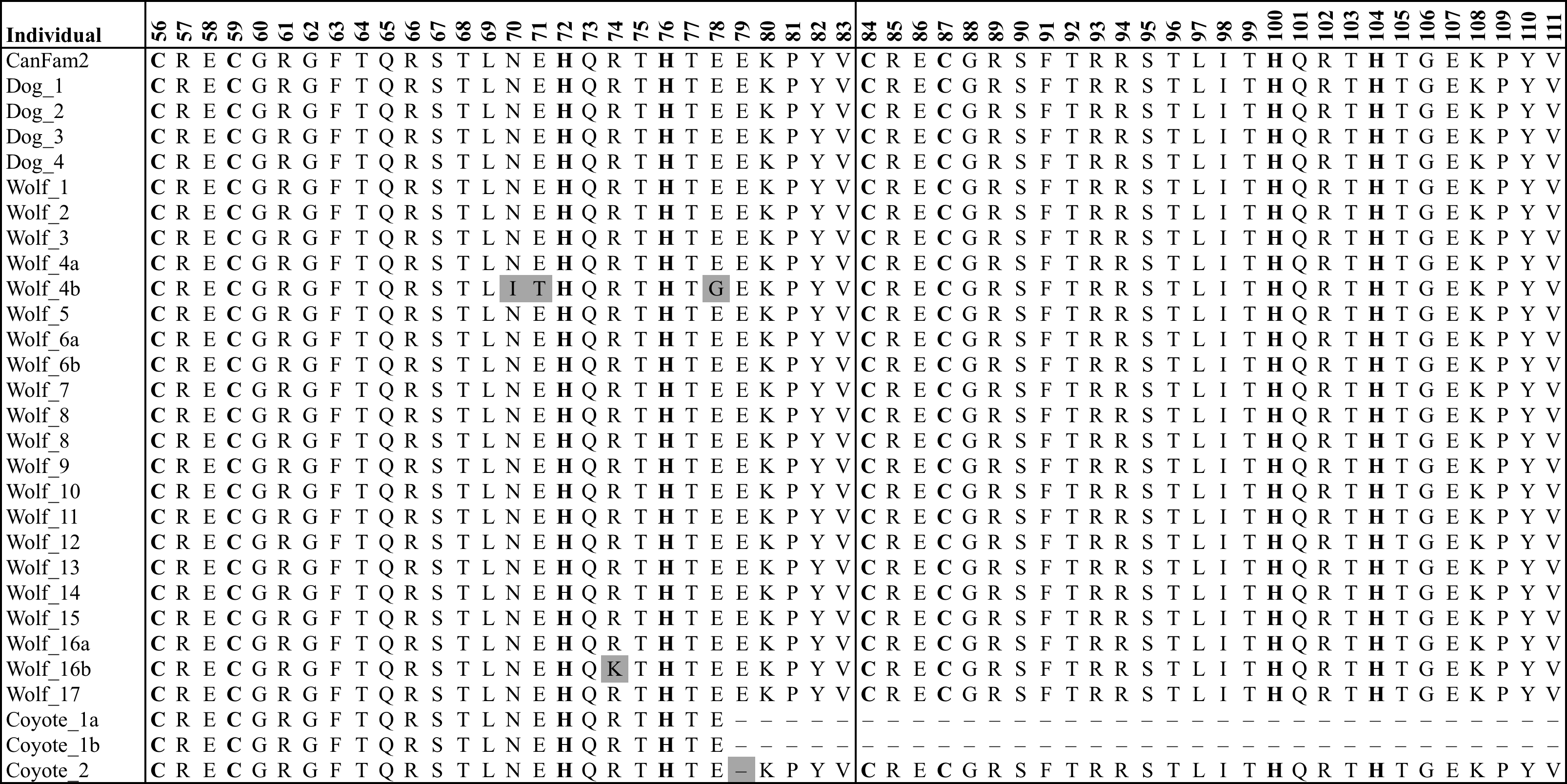

Supplement: Table S1 — The four C2H2 ZF-like regions found in the three canid species studied (dogs, wolves and coyotes). (DOC) [file pone.0025498.s001.doc]
