# Supplementary material for: Prdm9, a Major Determinant of Meiotic Recombination Hotspots, Is Not Functional in Dogs and Their Wild Relatives, Wolves and Coyotes
Source: PLoS One. 2011 Nov 10;6(11):e25498. doi: 10.1371/journal.pone.0025498 (PMC3213085; doi:10.1371/journal.pone.0025498)

***Prdm9*, a Major Determinant of Meiotic Recombination Hotspots, Is Not Functional in Dogs and Their Wild Relatives, Wolves and Coyotes**

**Violeta Muñoz-Fuentes, Anna Di Rienzo, Carles Vilà**

**SUPPLEMENTARY INFORMATION**

**Table S2. Translation to amino acids of the region in dogs, wolves and coyotes aligning to the PRDM9 region identified by [17] as conserved across 15 mammal species.**

This region is located upstream of the ZF domain. For comparison to our data, here we show the cat (*Felis catus*) sequence for being the species phylogenetically closest to the dog among those in the original study, and the one corresponding to the human (*Homo sapiens*). Stop codons are represented by an asterisk with a square around them and were observed in all reading frames for the three canid species; here we report the first reading frame, following the one used by [17]. All stop codons are at the same position in the three canid species studied (24 individuals). Identical amino acids found at the same position in the human, the cat and the canids are indicated in light grey; for clarity, this is shown only for the first canid in the table. A dot indicates an identical amino acid to the one in the dog genome sequence, CanFam2.


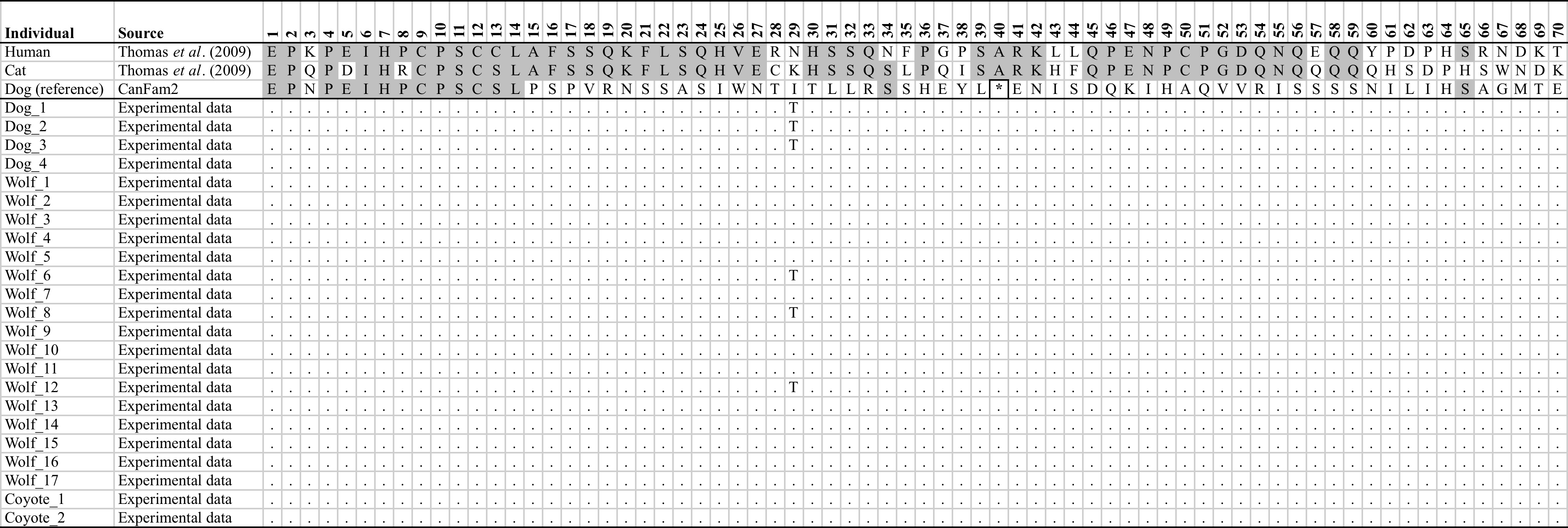


**Table S2 (continued)**


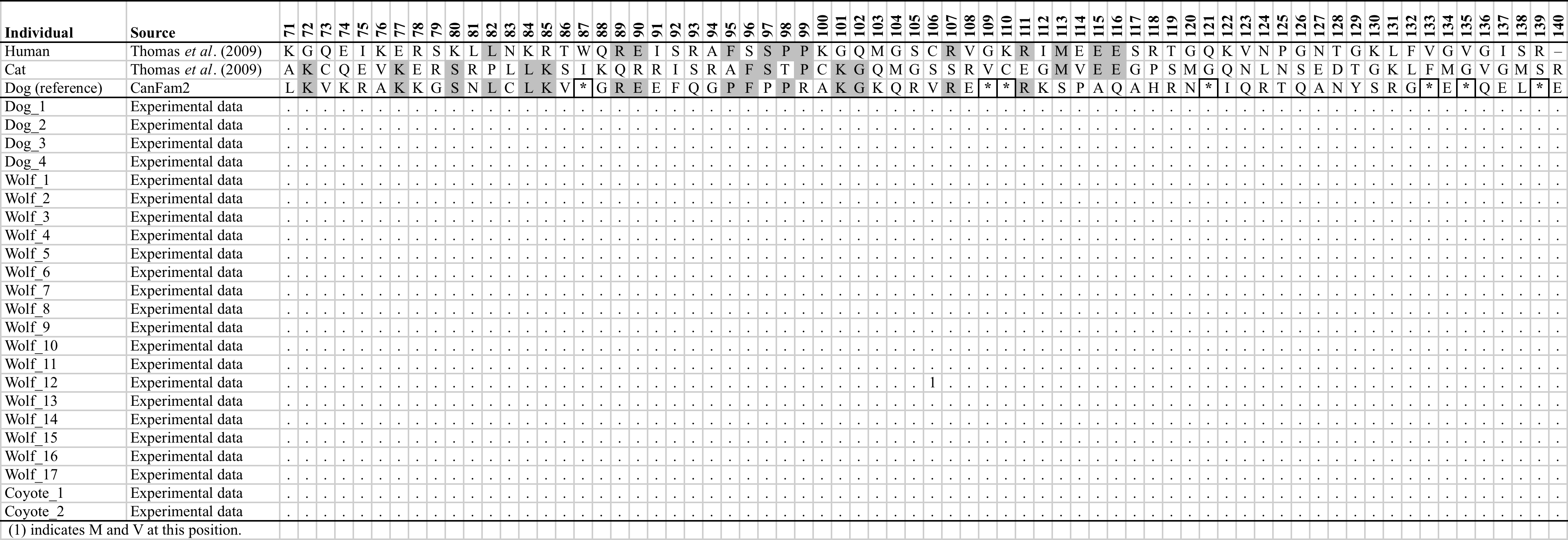

Supplement: Table S2 — Translation to amino acids of the region in dogs, wolves and coyotes aligning to the PRDM9 region identified by [17] as conserved across 15 mammal species. (DOC) [file pone.0025498.s002.doc]
